# Evaluation of the Influence of Adalimumab on the Expression Profile of Leptin-Related Genes and Proteins in Keratinocytes Treated with Lipopolysaccharide A

**DOI:** 10.3390/ijms22041595

**Published:** 2021-02-05

**Authors:** Beniamin Oskar Grabarek, Tomasz Kasela, Iwona Adwent, Barbara Zawidlak-Węgrzyńska, Ryszard Brus

**Affiliations:** 1 Department of Histology, Cytophysiology, and Embryology in Zabrze, Faculty of Medicine in Zabrze, The University of Technology in Katowice, 41-800 Zabrze, Poland; iwonaadwent7@gmail.com; 2 Department of Nursing and Maternity, High School of Strategic Planning in Dąbrowa Górnicza, 41-300 Dąbrowa Górnicza, Poland; hrbrus@wp.pl; 3 European Center of Aestheticsin Katowice, 40-055 Katowice, Poland; kaselatomasz@gmail.com; 4 Department of Chemistry in Zabrze, Faculty of Medicine in Zabrze, The University of Technology in Katowice, 41-800 Zabrze, Poland; barbara.zawidlak@wst.com.pl

**Keywords:** leptin, adalimumab, keratinocytes, lipopolysaccharide A, mRNA, siRNA, signaling pathway

## Abstract

Psoriasis is a disease with a proinflammatory base, in which an increased expression of leptin, tumor necrosis factor alpha (TNF-α), interleukin (IL) IL-12/23, IL-6, is observed. A drug used in the treatment of psoriasis of moderate and acute strength is the monoclonal antibody anti-TNF–adalimumab. The goal of this study was to evaluate the influence of adalimumab on changes in the expression profile of leptin-related genes in human keratinocyte cells exposed to lipopolysaccharide A and analyze if adalimumab acts via leptin pathways. The evaluation of changes of the pattern of genes connected with leptin and proteins coded by them was marked in a culture of human keratinocytes (HaCaT) exposed to 1 µg/mL lipopolysaccharide A (LPS) for 8 h in order to induce the inflammatory process, then to 8 µg/mL of adalimumab for 2.8 and 24 h in comparison with the control (cells not treated with the substances). The techniques used were mRNA microarray, Real-Time Quantitative Reverse Transcription Reaction (RTqPCR), Enzyme-Linked Immunosorbent Assay (ELISA), as well as transfections of HaCaT culture with leptin small interfering RNA (siRNA) in order to see whether adalimumab works through pathways dependent on leptin. A statistically lower expression of leptin and its receptors was observed under the influence of the drug, independent of the exposition time of keratinocytes to adalimumab. In the cells transfected with leptin siRNA, a lower concentration of JAK2 and STAT3 proteins was observed, which confirms that adalimumab works through pathways dependent on leptin. Adalimumab has a modulatory effect on the gene expression pattern and the proteins coded by them connected with leptin in keratinocytes treated with LPS in vitro.

## 1. Introduction

Leptin (LEP) is an adipokine, a protein with a mass of approximately 16 kDa, whose expression takes place for the most part in the cells of the fatty tissue described as adipocytes. The source of leptin in circulation is mostly fatty and visceral tissue [1]. The strengthening of LEP mRNA expression and the secretion of this adipokine into the blood is observed with a higher concentration of insulin and glucocorticosteroids, while the silencing of this expression is induced by an increase in the concentration of glucagon and catecholamines [2,3]. The biosynthesis of leptins in adipocytes of the fatty tissue is determined by the mass of this tissue and the size of the cells which make it up. It was observed that in fatty tissue cells of obese people the expression of leptin mRNA was higher than in the fatty tissue cells of slender people [4,5]. In the obese group, there is a significantly higher concentration of leptin in the serum, with values as high as 30 ng/mL, while on the other hand in the slender group with the correct body mass index (BMI), the average concentration of the aforementioned adipokine oscillates between 2 and 8 ng/mL [6,7].

Leptin influences the target cells by modulating simultaneously defined biological processes by binding them with adaptive receptors: leptin overlapping transcript (LEPROT), leptin receptor overlapping transcript-like 1 (LEPROTL1), and leptin receptor (LEPR) [7,8]. As a result of the aforementioned interaction, signaling pathways are activated, among which the most important are the JAK/STAT pathway, as well as the protein kinase signaling pathways activated by mitogens (MAPK) [9,10].

Cells that are able to express receptors for leptin include hypothalamus cells, mononuclear peripheral blood cells, vascular endothelial cells, fibroblasts, and keratinocytes. Leptin plays an important role in immune processes. It was observed, that under the influence of adipokine, monocytes and macrophages are activated, as a result strengthening the secretion of proinflammatory cytokines e.g., tumor necrosis factor-alpha (TNF-α), and interleukin (IL) IL-6, IL-9, IL-12, and IL-23, as well as the differentiation of naive T lymphocytes to the Th1 phenotype [11,12]. Moreover, leptin is characterized by proliferative properties in relation to keratinocytes [13].

Apart from an undeniable connection between an increased concentration of leptin and obesity [1,2,3], an excessive expression of this adipokine is observed in psoriasis, which is a disease with a proinflammatory base, during which the balance between pro- and anti-inflammatory cytokines is disturbed. A characteristic symptom of dermatosis is the phenomenon of parakeratosis, meaning an 8-times faster epidermal keratinization [14]. In a study conducted by Hamming et al., it was shown that an increased level of leptin in obese patients may be significant in the etiopathogenesis of psoriasis, which they connected with leptin inducing specific signaling pathways, of which the end products are TNF-α and IL-6 [15]. A fact which must also be taken into consideration is that psoriasis keratinocytes exhibit immunity to signals transmitted by TNF-α, which results in an increase in TNF-α concentration in skin alterations as well as serum in patients with psoriasis [16,17]. This was a reason for using TNF-α inhibitors in therapy of moderate and acute psoriasis. One of these is adalimumab which exhibits the ability to bind and inactivate membrane and soluble form of TNF-α [18], which in turn, as we have observed in previous studies, has an effect on the JAK/STAT cascade [19]. In in vitro studies in order to replicate inflammatory conditions in keratinocytes altered by psoriasis, lipopolysaccharides A are used [20]. However, the knowledge on the role of leptin in keratinocytes is incomplete.

The goal of this study was to evaluate the influence of adalimumab on changes in the expression profile of leptin-related genes in human keratinocyte cells exposed to lipopolysaccharide A and analyze if adalimumab acts via leptin pathways.

## 2. Results

### 2.1. LPS and Adalimumab XTT Cytotoxicity Assay

**Figure 1:**
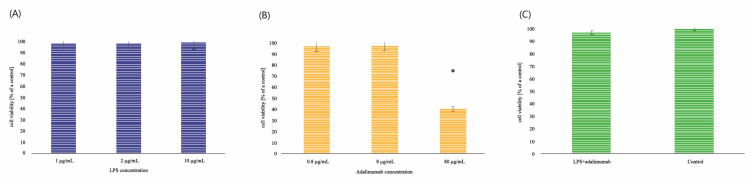
Results of XTT cytotoxicity assay HaCaT with LPS (**A**); HaCaT with adalimumab (**B**); HaCaT with LPS and adalimumab in comparison to the control (**C**). * statistically significant differences in comparison to the control (*p* < 0.05)**.**

The XTT cytotoxic test did not indicate that lipopolysaccharide A (LPS), adalimumab, or a combination of them in keratinocytes in the concentration chosen to analyze the expression pattern of leptin-related genes affect the vitality of these cells (*p* > 0.05, one-way ANOVA test). In turn, the statistical analysis showed statistically significant differences between HaCaT exposed to 80 µg/mL and untreated cells (*p* < 0.05). When the highest concentration of adalimumab was used, it was observed that only 40.64% of cells remained alive in comparison to the control culture (*p* < 0.05). The results of the XTT cytotoxicity assay are shown in Figure 1A–C.

### 2.2. Assessment of Caspases-3, -8 and -9 Activity in the HaCaT Cell Line Treated with Adalimumab after Inducing Inflammation by LPS

**Figure 2:**
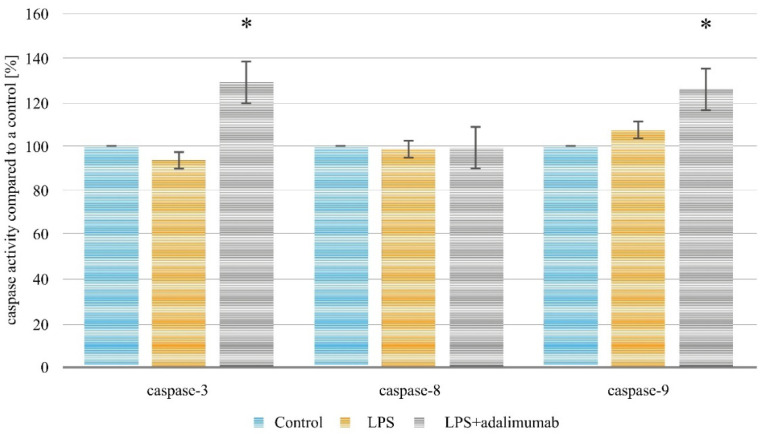
Caspases-3, -8, and -9 activity in HaCaT cell line exposed to 1 µg/mL LPS and then 8 µg/mL (* statistically significant differences in comparison to the control cell culture; *p* < 0.05; control-cells treated with PBS; 100%).

To evaluate the proapoptotic properties of 8 µg/mL adalimumab in keratinocytes which had been inflamed with 1 µg/mL LPSP, commercially available assays to determine the level of three caspases were used. The statistical analysis showed that changes in caspases-3 and -9 activity were significant (*p* < 0.05). It was observed that activity of caspase-3 was higher in the culture with adalimumab in comparison to the control culture about 29.04 ± 1.07% (*p* = 0.0000), while for caspase-9 there was an increase of about 25.77 ± 1.19% in HaCaT treated with the anti-TNF drug compared to untreated cells (*p* < 0.05). In turn, statistically significant differences were not observed in caspase-8 activity in comparison cell cultures (*p* > 0.05). The results of the test are shown in Figure 2.

### 2.3. Expression Profile of Leptin-Related Genes in HaCaT Culture Exposed to LPS, LPS and Adalimumab and in a Control Culture as Determined by Microarray, RTqPCR

Out of 38 mRNAs associated with leptin pathways, the highest differences in the expression pattern in the keratinocyte cell line exposed to LPS, LPS and adalimumab in comparison to the control culture (untreated cells) were observed for leptin and its receptors (*p* < 0.05). It can be seen that, in the cell culture exposed to LPS, the transcriptional activity of leptin and its receptors is statistically higher than in the control culture of keratinocytes (*p* < 0.05). However, under the influence of adalimumab, even at the shortest time of exposure to the drug, a decrease in the expression of the analyzed genes was noted, both in comparison with cells where an inflammatory condition was induced with the aid of LPS as well as those which were untreated (*p* < 0.05). Changes in the profile of expression of leptin-coding genes and receptors observed in the microarray experiment were confirmed using the RTqPCR method (Table 1).

### 2.4. The Level of Leptin (Protein) in Keratinocytes Exposed to LPS, and Adalimumab in Comparison with the Control Culture

**Figure 3:**
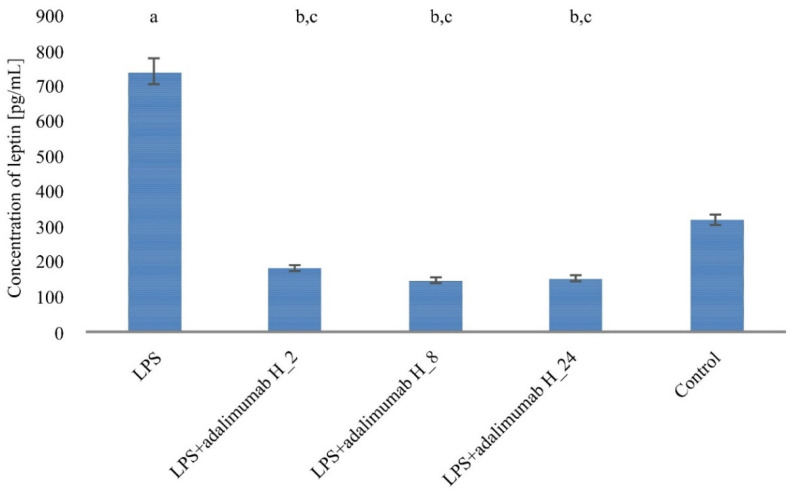
Changes in the expression of leptin in the HaCaT cell line treated with LPS, LPS and adalimumab, compared to the control culture (untreated cells) obtained by the ELISA assay (a—statistically significant differences in the expression of leptin between cells exposed to LPS vs. control *p* < 0.05; b—statistically significant differences in the expression of leptin between cells exposed to LPS and adalimumab vs. control *p* < 0.05; c—statistically significant differences in the expression of leptin between cells exposed to LPS and adalimumab vs. cells exposed to LPS *p* < 0.05).

In the culture of appropriate keratinocytes exposed to 1 µg/mL LPS for 8 h a statistically higher concentration of leptin was observed, in comparison to the control culture (742.4 ± 19.07 pg/mL vs. 321.04 ± 14.75 pg/mL; *p* < 0.05). Moreover, in the case of keratinocyte culture initially incubated with LPS in a concentration of 1 µg/mL, to which 8 µg/mL adalimumab was later added and cells were incubated for 2.8 and 24 h in comparison with keratinocytes untreated with LPS and adalimumab, the leptin concentration was statistically lower H_2 vs. C 185.04 ± 11.08 pg/mL vs. 321.04 ± 14.75 pg/mL; *p* < 0.05; H_8 vs. C 149.09 ± 12.55 pg/mL vs. 321.04 ± 14.75 pg/mL; *p* < 0.05; H_24 vs. C 154.25 ± 24.74 pg/mL vs. 321.04 ± 14.75 pg/mL; *p* < 0.05; Figure 3).

### 2.5. RNA Interference Analysis

The last step of our study was to examine the level of JAK-2 and STAT3 proteins in the HaCaT culture treated with LPS and adalimumab and transfected with leptin siRNA or scramble control siRNA by the ELISA assay method. Cells incubated for 0 h with LPS and the drug constituted the control culture at this stage of molecular analysis. The results indicated that leptin siRNA inhibited the expression of JAK2 and STAT3 proteins in HaCaT untreated with LPS and the drug and in the culture exposed to LPS and adalimumab for 2.8 and 24 h.

**Figure 4:**
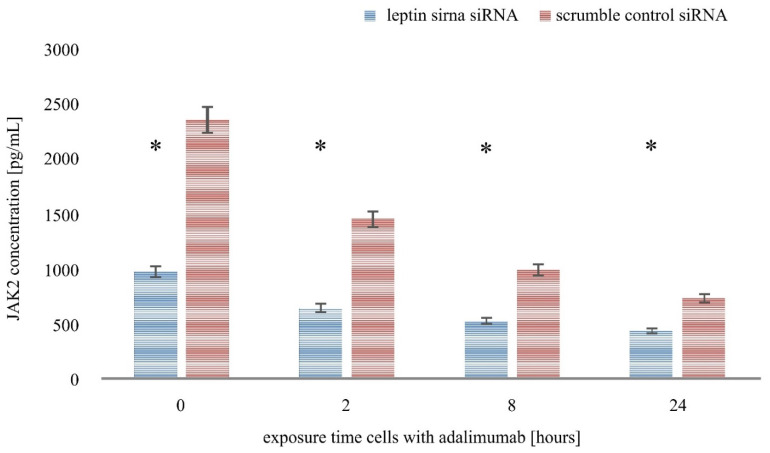
Leptin siRNA decreases JAK2 expression in the human keratinocyte cell line treated with LPS and adalimumab, and in the control culture. *—statistically significant differences *p* < 0.05.

**Figure 5:**
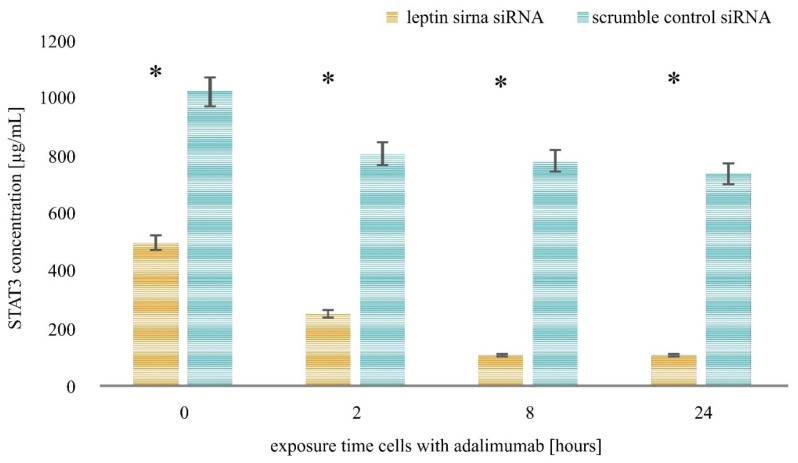
Leptin siRNA decreases STAT3 expression in the human keratinocyte cell line treated with LPS and adalimumab, and in and in the control culture. *—statistically significant differences *p* < 0.05.

Taking into consideration the changes in the expression profile of the selected proteins in the HaCaT culture with leptin siRNA and the culture with a scramble control siRNA (a negative control of the experiment), it can be noticed that adalimumab intensifies the effect of leptin siRNA. Statistical analysis indicated that regardless of the incubation time of the HaCaT cell line with the siRNA vector, the variances in the expression profile of JAK2 and STAT3 were statistically significant compared with cells treated with the scramble control siRNA (Figure 4 and Figure 5; *p* < 0.05).

## 3. Discussion

Studies concerning the molecular mechanisms of induction and development of psoriasis as well as factors connected with obtaining an adequate response to treatment have been conducted for some time. They focus, not only on attempting to point out new molecular markers useful in the diagnosis of dermatosis or constituting new molecular goals but also on the analysis of mutual bonds between cytokines and the growth factor [21,22].

A significant role in psoriasis is also attributed to cytokines released by cells of the fatty tissue, known as adipokines [23]. There are several reasons to evaluate the changes of gene pattern, connected with signaling pathways activated by leptin. Firstly, numerous findings confirm a significantly higher concentration of leptin among psoriasis patients, especially in its moderate and acute form [24,25], in which treatment with anti-cytokine agents is recommended [18]. Secondly, a positive correlation was noted between the concentration of leptin in the serum and BMI [26,27], while the influence of leptin on receiving the response to the applied treatment and maintaining the period of disease remission, was also determined [28].

In the present work, we decided to evaluate the way that adalimumab influences the expression of leptin-related genes as well as the process of proliferation and apoptosis in keratinocytes, in which an inflammatory condition had been previously induced by adding LPS to the medium of the culture.

During the course of psoriasis, there is a halt in proapoptotic processes in keratinocytes with their simultaneous hyperproliferation. For this very reason, the aim of the therapies used in psoriasis is to activate processes of programmed death of keratinocytes or halt their proliferation [29]. Keratinocyte proliferation, which is induced and then strengthened by cytokines, growth factors, and chemotactic factors, contributes to epidermal hyperplasia, hyperkeratosis, and parakeratosis [30,31]. Here, the mechanism of the vicious cycle of the self-strengthening inflammatory condition can be observed, since, e.g., the secretion of some cytokines accelerates the expression of others [32].

In our work, the evaluation of the influence of adalimumab on the apoptosis of keratinocytes with an induced inflammatory condition was defined by the analysis of caspases-3, -8, and -9. Caspases are cysteine proteases, biosynthesized in the form of enzymatically inactive proteins—zymogenes. There are three groups of enzymatic proteins. The first includes caspases-1, -4, -5, -11, -12, -13, and -14, mostly connected with the process of activation of cytokines which take part in the inflammatory process. The second group is referred to as effector caspases, which include caspases-3, -6, and -7. Finally, the third group of initiator caspases is made up of caspases-2, -8, -9, -10, and -12 [33,34].

It should be noted, that the exposition of keratinocyte cells to adalimumab in a statistically significant way increases the activity of caspases-3 and -9 in comparison with the control culture. The activation of caspase-8/-10 activates a cascade of caspases connected with the proteolysis of enzymes and structural proteins of cells and is characteristic of the extrinsic pathway of apoptosis (independent of mitochondria), beginning the proteolysis of enzymes and cellular structure proteins which occurs in cascades. On the other hand, the key role in the intrinsic pathway (otherwise known as the mitochondrial pathway) is played by caspases-3, -6, -7 [35]. Therefore, it seems that adalimumab induces apoptosis through the pathway dependent on mitochondria [36].

Shen et al. confirm the increase of caspase-3 activity under the influence of adalimumab using the human monocytic cell line model, as well as the chimeric human-mouse model. The changes in the cell culture model were noted by these scientists as early as 1 h after the incubation of the cells with the drug [37], which is consistent with our observations. On the other hand, Paula et al. did not show a statistically significant difference in the expression of caspases-8 and -9 mRNA, when adalimumab was added to the animal experimental model of retinal cells in comparison with the placebo and the control [38]. The differences between the results presented by Paula et al. [38] and our own may result from either using different study models or from the fact that the retinal cells prior to exposition to adalimumab were not stimulated with a proinflammatory factor.

Adalimumab is a fully human monoclonal antibody that neutralizes the soluble and transmembrane form of TNF-α. This neutralization only takes place before the occurrence of the interaction between TNF-α and its receptors—TNFR1 and TNFR2. Therefore, it seems that in the situation where the aforementioned interaction occurs, the effectiveness of adalimumab is limited [39]. In a previous study of ours we indicated that adding adalimumab to a culture of regular skin fibroblasts does not cause a complete neutralization of TNF-α. Hence, this drug may act at different stages of signal transduction [40]. Reinartz et al. indicated that HaCaT proliferation was reduced and apoptosis was induced in cells treated with TNF-α [41]. Our results indicated that adding LPS keratinocytes and adalimumab does not have a cytotoxic effect, with simultaneous activation of caspases-3 and -9. However, it should be noted that the result of the XTT test (Figure 1A) did not indicate that the adalimumab added to the culture of LPS keratinocytes caused an increase in cell proliferation, which would be paradoxical. On the other, no statistically significant decrease in cell viability was noted. Therefore, taking into account together the results of the XTT test and the activity of caspases can indicate an activation of adaptive mechanisms, through which despite an increase in the activity of caspases-3 and -9, a decrease in the proliferation of keratinocytes during research was not noted [42,43]. In a subsequent stage, after showing that LPS and adalimumab in selected concentrations do not induce a cytotoxic effect towards keratinocytes, the expression of leptin and its receptors on the level of transcriptome and proteome was defined. A statistically significant increase in the expression of the evaluated genes occurred after adding LPS to the cell culture, which confirms the fact that in an inflammatory condition the concentration of leptin and its receptor is at a higher level than during physiological conditions. Then, after adding adalimumab to the HaCaT culture, a statistically significant decrease in the expression of the evaluated genes was noted. Lee et al. attempted to evaluate the influence of leptin on normal human keratinocytes (NHK) by determining a microarray profile of expression, whose activity differentiates the culture incubated with leptin in comparison with the control. They observed that 151 transcripts are characterized by a higher expression, while 53 by a lower expression in the NHK culture with leptin (*p* < 0.05), while the biological effects were exerted through the JAK/STAT pathway [44]. Similarly, the observations made by Strejnholm et al., on a commercially available cellular line of keratinocytes, confirmed that leptin is an indicator of the inflammatory process and increases the proliferation of epidermal cells [45].

Therefore, in the case of diseases with underlying proinflammatory conditions which include psoriasis [14,30,32] it is important to lower the level of expression of leptin and genes connected with it. In their work, Emilio-Silvia et al. show that a mixture of terpenes in the form of citral lowers the concentration of leptin and IL-6, TNF-α in the serum of mice, where a systemic inflammatory response was induced by LPS [41]. These results confirm the fact that the JAK/STAT signaling pathway, whose final products are TNF-α and IL-6, is a key signaling cascade activated by the interactions between leptin and its receptors [19,46].

However, treatment, in which one of the effects is the decrease in the concentration of leptin must be very precise and must suit each patient [47]. This is important since it was noted that leptin, apart from the already described negative influence [2,3,13,24,25], also has a protective effect, e.g., it protects against depression [48]. Hernández et al. conducted a study on a group of 29 patients with moderate to acute psoriasis treated with adalimumab. The first dose of the drug equaled 80 mg, then every following week, the patients were given 40 mg of it. The whole study lasted for 18 months. It was observed that after a 6-month anti-TNF therapy, the concentration of leptin in the patients’ serum was significantly lower than prior to the beginning of treatment [49]. On the other hand, meta-analysis performed by Kyriakou et al. suggests a lack of the influence of an anti-cytokine therapy on the expressive profile of leptin. However, these authors do not wholly negate the possibilities of changes in the concentration of leptin under the influence of drugs used in psoriasis, at a time when the weight of the patients also decreases [50]. Nevertheless, the analysis of the influence of adalimumab conducted within the framework of the present work, indicates that this anti-TNF drug also exerts its influence through signaling pathways connected with leptin. With this in mind, the leptin siRNA vector or scramble control siRNA was added to a keratinocyte culture exposed to LPS and adalimumab and the control culture, and then the concentration of JAK2 and STAT3 proteins was marked using the ELISA method. It was observed, that in the culture transfected with leptin siRNA, the level of concentration of JAK2 and STAT3 proteins was statistically lower than in the control culture. Thus, it seems that the therapeutic effects exerted by adalimumab are also directly connected with the influence of this drug on genes and the proteins coded by them, connected with leptin. This further underlines the importance of molecular research in order to understand the etiology of diseases and mechanisms of drug functioning [51]. Gkalpakiotis et al. analyzed the influence of ustekinumab (an anti-IL12/23 drug) in a concentration of 1 and 5 µg/mL of adalimumab in a concentration of 5 µg/mL in comparison with a control culture of a THP-1 macrophage line on the expression of leptin and its receptors. They concluded an increase of expression on the level of mRNA and the protein of leptin receptor, regardless of the drug used, and a decrease in the expression of leptin only under the influence of the anti-IL12/23 drug [52]. It is the results regarding the expression of leptin and its receptor obtained by Gkalpakiotis et al. [47], which differ from ours, and which may be caused by a difference in the concentration of adalimumab used in the study as well as different study models.

The following stage, after the conducted observations on the in vitro model, would be an evaluation of an animal model with an inflammatory condition induced by LPS. As was indicated by Chueng et al., mirroring the inflammatory process occurring in humans onto an animal model is extremely difficult. Scientists place high hopes on the xenotransplantation model. However, currently the most commonly used animal model for psoriasis is imiquimod (IMQ)-induced mouse model due to it being easy to use and its low cost [53].

On the other hand, Bocheńska et al. point out that although animal models of the disease have contributed to a significant development of knowledge on the topic of the illness and its treatment, due to differences in, among others, human and animal skin, the currently used models due not allow for a complete mirroring of the environment of this dermatosis in humans [54].

It is also worth mentioning research done by Costa et al., who administered LPS to mice in the form of nebulization in order to evaluate early and late phases of acute lung injury [55].

Stjerholm et al. have, in addition, shown that in mice with a deficit of leptin, in which the inflammatory process was induced with the use of IMQ, the symptoms of psoriasis, meaning, erythema, infiltrations, and scales on the back, as well as inflammation of the ear skin, measured by ear thickness were less pronounced in this group in comparison with mice without leptin deficit. These observations indicate an important role in psoriasis [45].

The advantages of our study include conducting an analysis of the gene pattern expression connected with leptin on the level of mRNA using the method of oligonucleotide microarrays and RTqPCR keratinocytes, along with pointing out the pathway through which this process occurs. It was also confirmed that adalimumab exhibits specific biological effects, also through signaling pathways dependent on leptin. However, independent of the results obtained by us, which seem to be promising, it would be reasonable to conduct a similar analysis in vitro on a keratinocyte model exposed to other proinflammatory factors and then adalimumab, and then conduct an evaluation among psoriasis patients and a control group in vivo.

## 4. Materials and Methods

### 4.1. Cell Culture

As material in our work, human keratinocyte cells (HaCaT; CLS Cell Lines Service, Eppelheim, Germany) were used. The cell culture was carried out with the use of Dulbecco’s modified Eagle’s medium (DMEM; Sigma-Aldrich, St. Louis, MO, USA) supplemented with high glucose (4500 mg/L, Sigma-Aldrich, St. Louis, MO, USA), 10% fetal bovine serum (FBS; Sigma-Aldrich, St. Louis, MO, USA), penicillin (100 U/mL; Sigma-Aldrich, St. Louis, MO, USA), streptomycin (100 mg/mL; Sigma-Aldrich, St. Louis, MO, USA), and glutamine (2 mm; Sigma-Aldrich, St. Louis, MO, USA). The keratinocytes were incubated with a 5% CO_2_ enriched atmosphere and at a constant temperature of 37 °C.

### 4.2. XTT Cytotoxicity Assay

In order to assess the cytotoxicity effect of the LPS and adalimumab on keratinocytes, XTT (sodium 3′-[1- (phenylaminocarbonyl)-3,4- tetrazolium]-bis (4-methoxy6-nitro) benzene sulfonic acid hydrate)) assay (In Vitro Toxicology Assay Kit, XTT based; Sigma-Aldrich, St Louis, MO, USA) was performed in accordance with the manufacturer’s protocol. The principle of the XTT test is based on the reduction of yellow tetrazole salt by mitochondrial dehydrogenases to orange formazan, the released amount of which is directly proportional to metabolically active (living) cells.

In order to determine the effect of these compounds, HaCaT were treated with lipopolysaccharide A (LPS; Sigma Aldrich, Poznań, Poland) in three different concentrations: 1, 2, and 10 μg/mL (induction of inflammation), and adalimumab (0.8, 8, and 80 μg/mL) in comparison with the untreated cells (control of the experiment). Taking into account, our previous studies, keratinocytes were incubated with 1 μg/mL LPS for 8 h [20] and next with 8 μg/mL adalimumab. The concentration of this drug corresponds to the concentration in the serum of psoriatic patients [19].

Based on the obtained absorbance readings, the percentage of the value obtained for control cells (100%) was determined to be the calculated absorption value for the individual salinomycin concentrations. The absorbance results from untreated cells (control) were described as 100% and this value was used to calculate the absorption value for individual LPS, adalimumab concentrations. The percentage of viable cells in the culture treated with LPS, adalimumab was indicated in comparison to the untreated cells.

### 4.3. Caspases-3, 8, and -9 Activity

The activity of caspases-3, -8, and -9 in the HaCaT culture exposed to 1 µg/mL LPS and 8 µg/mL adalimumab compared with the untreated cells was evaluated by using the caspases-8 and -9 activity assays (Caspase-8/Caspase-9 Colorimetric Assay Kit (R&D Systems, Minneapolis, MN, USA), caspase-3 activity was determined in cell lysates (EnzChek^®^ Caspase-3 Assay Kit #1 (Molecular Probes, Minneapolis, Willow Creek Rd, MN, USA) in accordance with the manufacturer’s recommendation. The measurement of the absorbance at λ = 405 nm (caspases-8 and -9) and λ = 520 nm (caspase-3) permitted the variances of caspase activity in cell lysates to be determined.

### 4.4. RNA Extraction

TRIzol reagent (Invitrogen Life Technologies, Carlsbad, CA, USA, Catalog Number 15596026) was used to extract the total ribonucleic acid (RNA) from the keratinocyte cell culture treated with LPS and adalimumab and a control culture as described in the manufacturer’s protocol. In order to purify extracts of RNA, the RNeasy Mini Kit (QIAGEN, Hilden, Germany, Catalog Number 74104) and DNase I enzyme (Fermentas International Inc., Burlington, ON, Canada, Catalog Number 18047019) were used. Next, extracts of RNA were diluted in 20 µL of sterile water and frozen at −70 °C until the time when the molecular analysis was made.

The last stage of RNA extraction was associated with evaluating the qualitative (1% agarose electrophoresis with 0.5 mg/mL ethidium bromide) and quantitative assessment (RNA concentration (wavelength 260 nm); purification by examining the absorption ratio; wavelength 260 nm/280 nm; standard 1.8–2.0; through the use of spectrophotometry).

### 4.5. Microarray Profile of Leptin-Related Genes

The evaluation of differences in the expression pattern of leptin-related genes in the HaCaT culture exposed to LPS and adalimumab in comparison with the control culture was determined by using the HG-U 133_A2 microarray (Affymetrix, Santa Clara, CA, USA and GeneChip™ 3′ IVT PLUS Reagent Kit and GeneChip™ HT 3′ IVT PLUS Reagent Kit (Affymetrix, Santa Clara, CA, USA, Catalog Number 902416) as described in the manufacturer′s protocol.

The microarray experiment can be included in the most critical stages:

1. Synthesis of double-stranded cDNA on the matrix of the ribonucleic extracts.
2. Adding the poly-A control and First Strand Master Mix to the total RNA, and next incubating it for 2 h at 42 °C.
3. Adding the Second Strand Master Mix to the reaction mixture and next incubating it for 1 h at 16 °C and next for 10 min at 65 °C.
4. Synthesis of biotinyl mRNA and incubation for 16 h at 40 °C.
5. Labeling mRNA with biotin and purifying,
6. Hybridization of mRNA specimens with microarray probes.
7. Reading of the hybridization signal and data analysis.

Out of 22,277 mRNA probes present on the microarray plate, 38 mRNAs were associated with leptin. The names of the probes were indicated on the Affymetrix NetAffx Analysis Center database after entering the query: “leptin” (http://www.affymetrix.com/analysis/index.affx; accessed on 1 October 2020). The Affymetrix Gene ArrayScanner 3000 7G and GeneChip^®^Command Console^®^Software were utilized for the analysis of fluorescence and intensity (Affymetrix, Santa Clara, CA, USA).

### 4.6. Real-Time Quantitative Reverse Transcription Reaction

In the next stage of molecular analysis, the data obtained from the microarray analysis was validated in the Real-Time Quantitative Reverse Transcription Reaction (RTqPCR). SensiFast SYBR No-ROX One-Step Kit (Bioline, London, UK) was used in accordance with the manufacturer’s protocol. Changes in the expression pattern of selected genes were presented as a fold change (FC) in comparison with the control culture (untreated cells). The 2^−∆∆Ct^ method was used, when FC for the control is equal 1.0. The reaction was carried out for genes for which the value of the fold change (FC) was the highest −4.0 ≤ FC ≥ 5.0.

The thermal profile of RTqPCR reaction was as following: reverse transcription (45 °C for 10 min); activation of the polymerase (95 °C for 2 min); 40 cycles including denaturation (95 °C for 5 s); annealing (60 °C for 10 s); elongation (72 °C for 5 s). The primer sequence of selected mRNAs was examined via RTqPCR and is shown in Table 2. β-actin (*ACTB*) was used as an endogenous control of the reaction.

### 4.7. Changes in the Concentration of Leptin

The penultimate part of the molecular evaluation was indicating the level of leptin on the proteome level by using the ELISA assay alongside the Adiponectin Human solid-phase sandwich Enzyme-Linked Immunosorbent Assay ELISA KIT (Life Technologies Corporation, Invitrogen, USA; Catalog Number; KAC2281). The ELISA assay consists of the following stages: binding to the antigen; adding the detector antibody; adding the IgG HRP and TMB substrate solutions; finally, stopping the solution according to the protocol. Next, the plate was read at 450 nm and a standard curve was generated to determine the concentration of leptin in the analyzed samples. Control was the untreated keratinocytes.

### 4.8. RNA Interference

In order to analyze the role of leptin, the effect of leptin knockdown on HaCaT cells, and to see if adalimumab exerts its effect via the leptin pathway, leptin siRNA was used. The keratinocyte cell line was exposed to 8 µg/mL adalimumab and/or 1 µg/mL LPS or without LPS and adalimumab was transfected with leptin siRNA (sequence 5′-CCAAAUAUCCAACGACCUG-3′) or scramble control siRNA (Dharmacon, Lafayette, CO, USA) using Lipofectamine2000 reagent (Thermo Fisher Scientific, Lipofectamine™ 2000 Transfection Reagent; Catalog Number 11668027) in accordance with the protocol. The culture was harvested 48 h after transfection. To evaluate whether the anti-TNF drug exerts its effect via the leptin pathway in the keratinocytes treated with LPS and adalimumab, siRNA was compared to the control culture, and the levels of JAK-2 (JAK2 ELISA Kit ab253224) and STAT3 (STAT3 ELISA KIT ab126427) were assessed by the ELISA assay.

### 4.9. Statistical Analysis

The licensed version of the Statistica 13.0 PL (StatSoft, Cracow, Poland) and the Transcriptome Analysis Console programs (Affymetrix, Santa Clara, CA, USA) were used in the statistical analysis. In the first step of the analysis, the normality of the data distribution using the Shapiro–Wilk test (*p* < 0.05) was examined. In the second stage, by using the ANOVA variance assay analysis, the differences shown were statistically significant, and in the last step, the post hoc Tukey′s test was also conducted (*p* < 0.05).

## 5. Conclusions

The conducted molecular analysis showed that adalimumab exerts a modulation effect on the gene pattern expression and the proteins coded by them connected with leptin in keratinocytes treated with LPS in vitro.

## Figures and Tables

**Table 1:** Variances in the expression pattern of leptin-dependent genes in HaCaT culture exposed to LPS or LPS+ adalimumab compared with the control-untreated cells (|FC| > 4.00; in the first comparison).

mRNA	ID	Microarray				RTqPCR			
		LPS	LPS + Adalimumab			LPS	LPS + Adalimumab		
		H-8 vs. C	H-2 vs. C	H-8 vs. C	H-24 vs. C	H-8 vs. C	H-2 vs. C	H-8 vs. C	H-24 vs. C
*LEP*	207092_at	+8.77 ^a^	−2.96 ^b^	−3.98 ^b^	−3.01 ^b^	+7.74 ^a^	−2.41 ^b^	−3.49 ^b^	−3.55 ^b^
*LEPROT*	202377_at 202378_s_at	+6.08 ^a^	−2.14 ^b^	−2.18 ^b^	−2.54 ^b^	+6.14 ^a^	−2.36 ^b^	−2.41 ^b^	−1.98 ^b^
*LEPROTL1*	202594_at 202595_s_at	+5.01 ^a^	+2.36 ^b^	−1.98 ^b^	−2.04 ^b^	+5.89 ^a^	+2.14 ^b^	−2.03 ^b^	−2.43 ^b^
*LEPR*	209894_at 209959_at 211167_s_at 211354_s_at 211355_x_at 211356_x_at	+4.18 ^a^	−3.55 ^b^	−3.10 ^b^	−2.11 ^b^	+4.22 ^a^	−3.64 ^b^	−2.74 ^b^	−1.56 ^b^

a—statistically significant differences in the expression of genes between cells exposed to LPS vs. control *p* < 0.05; b—statistically significant differences in the expression of genes between cells exposed to LPS and adalimumab vs. control (+) overexpression of gene (increased level of mRNAs); (–) suppressed gene expression (decreased level of mRNAs); FC—fold change; the 2^−∆∆Ct^ method; C—control (untreated cells; FC = 1); H_2, H_8, H_24—time exposure cells to LPS/adalimumab.

**Table 2:** Nucleotide sequence of primers used in the RTqPCR reaction.

mRNA	Nucleotide Sequence
*LEP*	Forward 5′-GAAGACCACATCCACACACG-3′ Reverse 5′-AGCTCAGCCAGACCCATCTA-3′
*LEPROT*	Forward 5′-GCTTGGAGAGGCAGATAACG-3′ Reverse ′-AATGTCCTGGGTCCAGAGTG-3′
*LEPROTL1*	Forward 5′-TGCAATGTGGGAAGAAATGA-3′ Reverse 5′-AAGGAGGAAGCAGAGGAAGG-3′
*LEPR*	Forward 5′-ACAGTCCCTTTGTGGGTCAG-3′ Reverse 5′-TATCCGAGCTCCAGCGTACT-3′
*ACTB*	Forward 5′-TCACCCACACTGTGCCCATCTACGA-3′ Reverse 5′-CAGCGGAACCGCTCATTGCCAATGG-3′

## Data Availability

The data used to support the findings of this study is included in the article. The data will not be shared due to the fact the third-party rights and commercial confidentiality.

## Abbreviations

RTqPCR	Real-time quantitative reverse transcription reaction
RNA	Ribonucleic acid
LPS	Lipopolysaccharide A
STAT	Signal transducer and activator of transcription
TNF-α	Tumor necrosis factor alfa
XTT	(2,3-Bis-(2-Methoxy-4-Nitro-5-Sulfophenyl)-2H-Tetrazolium-5-Carboxanilide)
ELISA	Enzyme-linked immunoassay
siRNA	Small interfering RNA
ACTB	β-actin
LEPROT	Leptin overlapping transcript
LEPROTL1	Leptin receptor overlapping transcript-like 1
LEPR	Leptin receptor
LEP	Leptin
MAPK	*mitogen-activated protein kinase*
IMQ	Imiquimod
